# Proteoglycan loss in the articular cartilage is associated with severity of joint inflammation in psoriatic arthritis—a compositional magnetic resonance imaging study

**DOI:** 10.1186/s13075-020-02219-7

**Published:** 2020-05-29

**Authors:** Daniel B. Abrar, Christoph Schleich, Sven Nebelung, Miriam Frenken, Tim Ullrich, Karl Ludger Radke, Gerald Antoch, Stefan Vordenbäumen, Ralph Brinks, Matthias Schneider, Benedikt Ostendorf, Philipp Sewerin

**Affiliations:** 1 University Dusseldorf, Medical Faculty, Department of Diagnostic and Interventional Radiology, D-40225 Düsseldorf, Germany; 2 Department and Hiller Research Unit for Rheumatology, UKD, Heinrich Heine University Düsseldorf, Moorenstrasse 5, 40225 Düsseldorf, Germany

**Keywords:** Psoriatic arthritis, Arthritis, Cartilage, Magnetic resonance imaging, dGEMRIC, Compositional imaging, Perfusion, PsAMRIS

## Abstract

**Background:**

Even though cartilage loss is a known feature of psoriatic arthritis (PsA), little is known about its role in the pathogenesis of PsA. Using delayed gadolinium-enhanced magnetic resonance imaging of cartilage (dGEMRIC) as a non-invasive marker of the tissue’s proteoglycan content, such early (i.e., pre-morphological) changes have been associated with inflammation in rheumatoid arthritis (RA). Yet, this association has not been studied before in PsA.

**Methods:**

The metacarpophalangeal (MCP), proximal interphalangeal (PIP), and distal interphalangeal (DIP) joints of 17 patients with active PsA were evaluated by high-resolution clinical standard morphological and dGEMRIC sequences using a 3T MRI scanner (Magnetom Skyra, Siemens) and a dedicated 16-channel hand coil. Images were analyzed by two independent raters for dGEMRIC indices, PsA MRI scores (PsAMRIS), and total cartilage thickness (TCT). Kendall tau correlation coefficients (*τ*) were calculated.

**Results:**

We found significant negative correlations between dGEMRIC indices and total PsAMRIS (*τ* = − 0.5, *p* = 0.012), synovitis (*τ* = − 0.56, *p* = 0.006), flexor tenosynovitis (*τ* = − 0.4, *p* = 0.049), and periarticular inflammation (*τ* = − 0.72, *p* < 0.001). Significant positive correlations were found between TCT and dGEMRIC indices at all joint levels (*τ* = 0.43, *p* < 0.001). No significant correlations were determined between dGEMRIC indices and bone erosion, bone edema, or bone proliferation.

**Conclusion:**

In PsA, proteoglycan loss as assessed by dGEMRIC is associated with periarticular inflammation, synovitis, and flexor tenosynovitis, but not with bone erosion or proliferation. Thereby, these findings contribute to in vivo concepts of the disease’s pathophysiology. Beyond morphology, advanced MRI techniques may be used to assess cartilage composition in PsA and to identify early changes in the cartilage as an imaging biomarker with potential application in detection, monitoring, and prediction of outcomes of PsA.

**Trial registration:**

2014123117, December 2014.

## Introduction

Psoriatic arthritis (PsA) is a chronic autoimmune disease that potentially leads to joint mutilation and high functional disability [1]. Similar to rheumatoid arthritis (RA), early diagnosis and treatment are crucial for the best clinical outcome [2, 3]. Consequently, treat-to-target (T2T) strategies are emerging for the treatment of PsA as in RA [4, 5]. Therefore, sensitive diagnostic tools are required for early diagnosis as well as monitoring of treatment efficacy and prediction of clinical outcomes. As a well-established imaging modality of contemporary medicine, magnetic resonance imaging (MRI) has become increasingly important in the clinical and scientific evaluation of rheumatic diseases. This is due to its high sensitivity for joint inflammation, even at subclinical stages, and joint damage as well as the close association of erosive progression of bone damage and MRI findings [6–8]. Recent trials, however, showed no significant superiority of an MRI-guided T2T strategy in RA [9–13]. As of today, it therefore remains unknown whether MRI-guided T2T strategies are beneficial in comparison with conventional disease activity-guided T2T strategies, even though personalized medicine holds great promise for improved health and healthcare, not least in RA [14]. The value of MRI is further emphasized by studies showing that it can predict therapy response and long-term outcomes in rheumatoid arthritis, even though concurring findings for PsA are yet to be determined [15, 16].

In 2009, the Outcome Measures in RA Clinical Trials (OMERACT) working group introduced a semi-quantitative PsA MRI score (PsAMRIS) that evaluates metacarpophalangeal (MCP), proximal interphalangeal (PIP), and distal interphalangeal (DIP) joints in terms of the osteodestructive (bone erosion), osteoproliferative (bone proliferation), and acute inflammatory features of PsA (synovitis, flexor tenosynovitis, periarticular inflammation, and bone edema) [12]. Even though cartilage damage is a known feature of PsA, unlike in RA, research is sparse on its role in the pathogenesis and course of the disease [11, 17]. Therefore, cartilage damage is not included in the PsAMRIS as opposed to its RA equivalent, RAMRIS, where the sub-score “joint space narrowing” reflects the degree of structural cartilage damage [18]. Research on cartilage-related biomarkers in PsA such as cartilage oligomatrix protein (COMP) and osteoprotegrin (OPG) indicates an involvement of the cartilage in the disease throughout all stages [19–21]. Additionally, studies have also demonstrated that cartilage involvement of the MCP joints in RA might originate at the entheseal insertion sites (i.e., the ligamento-cartilaginous junction), possibly due to mechanical stress and spreading inflammation [22]. Since PsA is known to be an entheseal-driven disease affecting the “synovio-entheseal complex,” a similar mechanism of cartilage damage has been suggested [23, 24]. In addition to mere morphological MRI techniques as implemented in PsAMRIS, several compositional MRI techniques are available that allow the detection and quantification of early cartilage changes on a molecular (and pre-morphological) scale [25]. Among these methods, delayed gadolinium-enhanced MRI of cartilage (dGEMRIC) is arguably the best validated and cross-referenced compositional MRI technique to visualize proteoglycan loss in the articular cartilage and has been successfully applied to identify cartilage damage in early RA despite the absence of relevant joint space narrowing [26–28]. Moreover, in RA, early cartilage changes determined by dGEMRIC are closely associated with acute joint inflammation measured by dynamic contrast-enhanced (DCE) MRI and the respective RAMRIS sub-score [29–31].

While for RA, a strong body of scientific evidence has linked dGEMRIC to the detection of (pre-morphological) cartilage damage and associated intra- and periarticular changes, these associations have not yet been studied for PsA. Against this background and on the basis of high-resolution morphological and compositional MRI techniques (i.e., dGEMRIC), the present study aimed to study the relationship of early cartilage damage and associated osteodestructive, osteoproliferative, and acute inflammatory changes (as sub-scores of PsAMRIS) at the MCP, PIP, and DIP joint levels in patients with long-standing PsA. Our hypotheses were (i) dGEMRIC maps may be used to visualize pre-morphological cartilage damage in PsA and (ii) dGEMRIC values are correlated with the global PsAMRIS (sum score) and its inflammatory, osteodestructive, and osteoproliferative sub-scores.

## Methods

### Study population

Twenty-one adult patients with PsA (mean age, 47 ± 6 years; range 26–72 years; male/female 11/10) fulfilling the CASPAR criteria and suffering from peripheral joint involvement of at least two MCP joints and dactylitis of at least one finger, but without nail involvement, were prospectively screened within the “Analysis of the DActylic Melange” (ADAM) research initiative [32]. All patients were receiving methotrexate (MTX) monotherapy. Of these 21 patients, 17 patients (mean age, 53.7 ± 11.6 years; range 26–72 years, male/female 9/8) were included for the present study. Their mean disease duration at the time of recruitment was 4 ± 3.6 years. The Disease Activity Score 28 (DAS 28) was 2.42 ± 0.72 (range 1.8–4.3, median 2.2). C-reactive protein (CRP) levels were 0.87 ± 1.35 mg/dl (range 0.1–5.8 mg/dl, median 0.3 mg/dl). All patients’ characteristics are summarized in Table 1. Patient recruitment took place in the Department of Rheumatology from June 2015 to January 2017.

**Table 1:** Patients’ characteristics. Patients’ age, sex, disease duration, serum levels of C-reactive protein (CRP), and disease activity score 28 (DAS-28) are presented. For each item—except sex—the mean ± standard deviation is given

Patient age	53.7 ± 11.6 years
Sex	9 males, 8 females
Disease duration	4 ± 3.6 years
CRP level	0.87 ± 1.35 mg/dl
DAS-28	2.42 ± 0.72

The study was approved by the local ethics committee (study number: 4962R, “Analysis of the Dactylitic Melange (ADAM): defining the morphological components of dactylitis in psoriatic arthritis and their responsiveness to etanercept therapy). Written and informed consent was obtained from all patients before the initiation of the study.

### MRI studies

Imaging studies were performed on a 3T MRI scanner (Magnetom Skyra, Siemens Healthineers, Erlangen, Germany) and a dedicated 16-channel hand coil (3T Tim receive-only Coil, Siemens Healthineers, Erlangen, Germany). Patients were imaged in the prone position with the hand and wrist extended overhead with the palm facing down.

The MRI protocol followed the recommendations of the OMERACT working group [12]. It included pre- and post-contrast T1-weighted and fat-saturated PD-/T2-weighted or short tau inverse recovery (STIR) and post-contrast fat-saturated T1-weighted images in at least two different planes. The field of view covered the MCP, PIP, and DIP joints 2–5.

Compositional MRI with dGEMRIC of the MCP, PIP, and DIP joints 2–5 was performed 40 min after intravenous (iv) administration of a gadolinium-based contrast agent (0.4 ml/kg body weight gadoteric acid [Gd-DOTA], Dotarem, Guerbet Villepinte, France). For T1 calculation, 3D gradient-echo imaging with two flip angles, 5° and 26° (termed 3D fast-low-angle-shot [3D-FLASH]), was acquired on a total of 40 sagittal slices that were oriented perpendicular to the joint spaces. The acquisition time for the 3D FLASH sequence was 2.25 min.

The detailed sequence parameters were as follows: coronal T1 TSE (turbo spin echo) sequence (TR/TE 862/27 ms, flip angle 150°, slice thickness 2.5 mm, field of view 140 × 140 mm, imaging matrix 512 × 512, pixel size 0.3 × 0.3 mm), coronal STIR (TR/TE, 5560/31 ms, flip angle 120°, slice thickness 2.5 mm, slice thickness 3.0 mm, field of view 140 × 140 mm, imaging matrix 448 × 312, pixel size 0.3 × 0.3 mm), sagittal proton density (PD) TSE with fat saturation (TR/TE 3150/47 ms, flip angle 150°, slice thickness 2.5 mm, field of view 60 × 150 mm, imaging matrix 448 × 182, pixel size 0.3 × 0.3 mm), transversal T2 TSE with fat saturation (TR/TE 5694/89 ms, flip angle 180°, slice thickness 3.0 mm, field of view 160 × 160 mm, imaging matrix 512 × 358, pixel size 0.3 × 0.3 mm), transversal T1 TSE with fat saturation after iv contrast administration (TR/TE 807/16 ms, flip angle 90°, slice thickness 3.0 mm, field of view 130 × 130 mm, imaging matrix 384 × 288, pixel size 0.3 × 0.3 mm), coronal T1 TSE after iv contrast (TR/TE 862/27 ms, flip angle 150°, slice thickness 2.5 mm, field of view 140 × 140 mm, imaging matrix 512 × 512, pixel size 0.3 × 0.3 mm), and 3D FLASH GE (TR/TE 5.8/1.9 ms, flip angle 5°/26°, slice thickness 3.0 mm, field of view 65 × 110 mm, imaging matrix 384 × 228, pixel size 0.3 × 0.3 mm).

### Image analysis

MR images were independently read and analyzed by two radiologists (DBA and CS, trained in musculoskeletal imaging with 3 and 8 years of experience, respectively) and one rheumatologist (PS, trained in musculoskeletal imaging with 8 years of experience) according to the OMERACT PsAMRIS guidelines [12]. Further, inflammatory changes of the extensor tendons and their surrounding tissue, i.e., “extensor tenosynovitis,” were quantified (scores 0–3), in analogy to the sub-score “flexor tenosynovitis.” The score reflected the maximum degree of enhancing and/or hyperintense signals within or surrounding the extensor tendon at its most inflamed part, and scores indicated the absence of any abnormality (score 0), the involvement of < 50% of the tendon (score 1), of ≥ 50 to < 100% (score 2), and ≥ its entire thickness (score 3). In addition to PsAMRIS, total cartilage thickness (TCT; sum of the total cartilage thickness of the articulating joint surfaces, i.e., the proximal and distal cartilage layers) was measured for each MCP, PIP, and DIP joint of fingers 2–5. One investigator (DBA) performed the thickness measurements perpendicular to the subchondral bone lamella in the ulnar, central, and radial third of the joint using the inbuilt digital caliper tool of the picture archiving and communication system (PACS, Sectra Workstation IDS7, Sectra AB, Linköping, Sweden) on sagittal PDW sequences. Subsequently, the mean thickness of all three measurements was calculated.

For compositional analyses of cartilage quality with dGEMRIC, motion correction was performed using STROKETOOL (Frechen, Germany) for all images to reduce movement artifacts. This tool has been validated for dGEMRIC analyses of the finger joints and corrects for patient motion between the measurements using a dedicated image registration method [33]. Readers were allowed to adjust the window settings as required to guarantee optimal visualization of the intra- and periarticular structures for ROI placement. T1 maps were analyzed by first defining the regions-of-interest (ROIs) on the central sagittal slice. ROI outlines comprising the full thickness of the proximal and distal portions of the articular cartilage of the MPC, PIP, and DIP joints 2–5 were manually defined on the morphological images of the 3D T1-weighted FLASH sequence with a flip angle of 5° for dGEMRIC. Particular care was taken to exclude artifacts and surrounding structures such as the synovial fluid and cortical bone. Consequently, four ROIs were set per digit (i.e., the metacarpal, the base of the proximal phalanx, the apex of the proximal phalanx, and the base of the intermediate phalanx) and 16 ROIs per patient (i.e., four ROIs of four digits) and visually checked by the second and third readers to confirm that only the cartilage was included. Next, ROIs were copied to the corresponding slices of the color-coded T1 parameter maps. Further analyses involved the pixel-wise calculation post-contrast T1 values as before [25, 31, 34]. More specifically, the T1 maps representing the spatially resolved dGEMRIC indices were analyzed in terms of the ROIs; as defined above, the mean dGEMRIC indices [ms] were recorded. All images were analyzed by two readers (DBA and CS, radiologists) who were blinded for patients’ data.

### Statistical analysis

All statistical analyses were performed using the SPSS software (IBM, version 22, Armonk, NY, USA). For descriptive analyses, the mean ± standard deviation, the range (minimum and maximum), and the median are presented in Tables 1, 2, and 3. Datasets were tested for normal distribution by the Kolmogorov-Smirnov test. Mean values were then compared with an analysis of variance (ANOVA) and a post hoc Scheffé test. For correlation analyses, the Kendall tau correlation was determined and quantified using the correlation coefficient *τ*. Correlation strength was graded as suggested by Cohen [35]: small (0.1–0.3), moderate (0.3–0.5), and large (> 0.5). *p* values < 0.05 were considered to be significant. Due to the explorative nature of the study, no correction for multiple testing was performed. For the evaluation of inter- and intra-rater reliability, single and average measure intraclass correlation coefficients (sICC and aICC) were calculated based on the dGEMRIC indices of the ROIs drawn by the two raters.

**Table 2:** Descriptive analysis of the psoriatic arthritis magnetic resonance imaging score (PsAMRIS) and sub-score values overall and at the MCP, PIP, and DIP joints 2–5 in PsA patients. For each item, the mean ± standard deviation is presented. Differences between joint levels were assessed for significance using one-way ANOVA. *p* values < 0.05 were considered significant and are given in bold

PsAMRIS	Joint level				*p* value
	Overall	MCP	PIP	DIP	
Total	67.47 ± 18	23.41 ± 4.89	22.94 ± 7.2	21.12 ± 9.71	0.456
Flexor tenosynovitis	10.47 ± 4.99	4.76 ± 1.44	2.94 ± 1.82	2.76 ± 2.31	**0.005**
Extensor tenosynovitis	12.24 ± 5.85	3.29 ± 2.68	4.94 ± 2.28	4.00 ± 2.72	0.127
Synovitis	22.12 ± 5.67	9.18 ± 2.1	7.41 ± 2.32	5.53 ± 2.43	**< 0.001**
Periarticular inflammation	19.76 ± 3.95	6.47 ± 1.66	6.88 ± 1.58	6.41 ± 1.66	0.144
Bone erosion	7.47 ± 5.46	2.29 ± 1.49	2.53 ± 2.83	2.65 ± 2.94	0.916
Bone edema	6.59 ± 5.47	0.59 ± 1.18	2.59 ± 2.24	3.41 ± 4.11	**0.015**
Bone proliferation	1.06 ± 1.39	0.12 ± 0.33	0.59 ± 0.87	0.35 ± 0.49	0.089

**Table 3:** Descriptive analysis of the total cartilage thickness (TCT) [mm] of the ulnar, central, and radial thirds of the MCP, PIP, and DIP joints 2–5 in PsA patients. For each item, the mean ± standard deviation, the median, and the range (minimum and maximum) are presented. Each TCT represents the sum of the proximal and distal cartilage layers of each joint. Means were compared by one-way ANOVA and a post hoc Scheffé test. *p* values < 0.05 were considered significant and are given in bold

TCT						
		Overall	MCP	PIP	DIP	*p* value
Ulnar	Mean	0.79	1.07	0.71	0.57	MCP vs. PIP **< 0.001** MCP vs. DIP **< 0.001**
	SD	0.33	0.30	0.23	0.22	
	Median	0.72	1.05	0.69	0.63	
	Minimum	0	0.5	0	0	
	Maximum	1.85	1.85	1.15	0.9	
Central	Mean	0.80	1.11	0.71	0.59	PIP vs. DIP **0.024** Ulnar vs. central 0.83
	SD	0.35	0.31	0.26	0.21	
	Median	0.74	1.07	0.68	0.66	
	Minimum	0	0.62	0	0	
	Maximum	2.18	2.18	1.41	0.98	
Radial	Mean	0.77	1.08	0.69	0.55	Ulnar vs. radial 0.989 Radial vs. central 0.751
	SD	0.32	0.25	0.23	0.21	
	Median	0.74	1.05	0.69	0.55	
	Minimum	0	0.55	0	0	
	Maximum	1.73	1.73	1.26	0.91	

## Results

### PsAMRIS and inflammation pattern

The results of the PsAMRIS and its sub-scores (flexor tenosynovitis, extensor tenosynovitis, synovitis, periarticular inflammation, bone erosion, bone edema, and bone proliferation) for all joints and the MCP, PIP, and DIP joints 2–5 are presented in Table 2. Despite differences in score ranges, inflammatory sub-scores, especially flexor tenosynovitis and periarticular inflammation, were higher than osteoproliferative or osteodestrutive sub-scores. Flexor tenosynovitis and synovitis sub-scores were significantly higher at the MCP joint level than at the PIP and DIP joint levels (flexor tenosynovitis: MCP vs. PIP *p* = 0.025, MCP vs. DIP *p* = 0.013; synovitis: MCP vs. PIP *p* = 0.001). The bone edema sub-score on the other hand was significantly lower at the MCP joint level compared to the DIP joint (*p* = 0.014). No significant differences were found between the three different joint levels regarding all other sub-scores or total PsAMRIS.

**Fig. 1:**
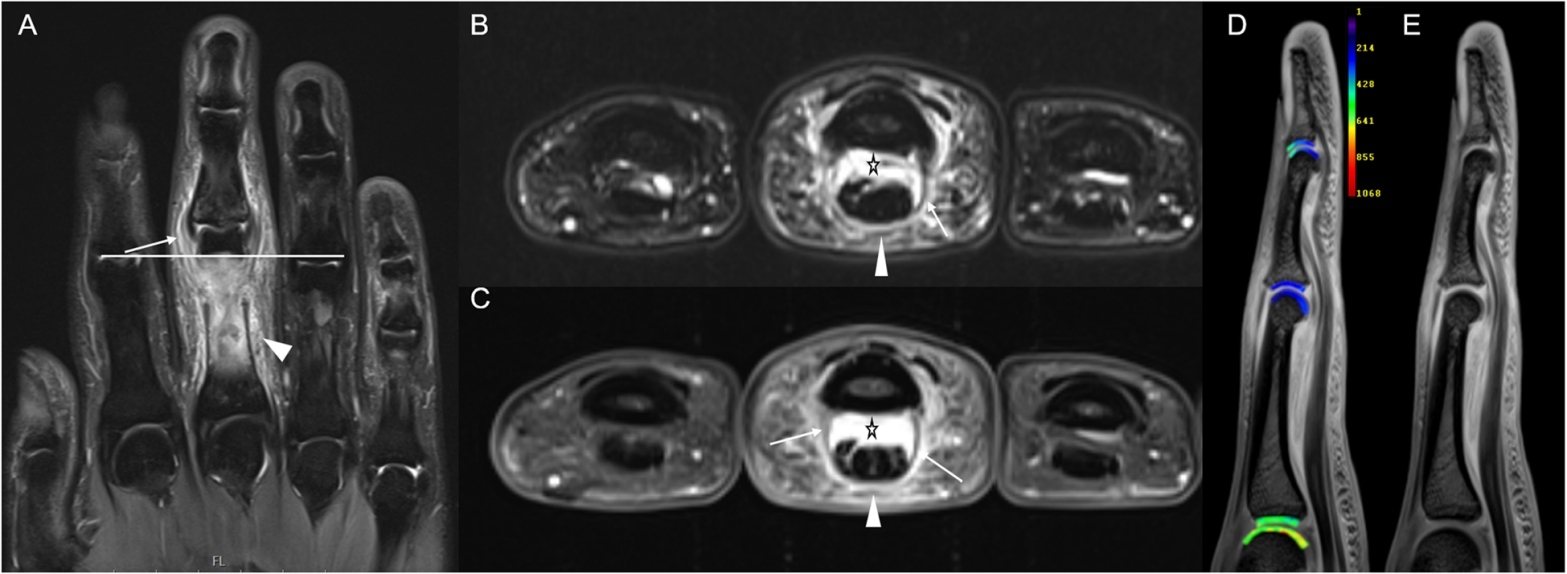
Right hand of a 26-year-old male with psoriatic arthritis (PsA; disease duration 39 months). Coronal STIR image (**a**) of digits 1–5, transversal fat-saturated (fs) T2-weighted image of digits 2–4 (**b**), and the corresponding transversal fs contrast-enhanced T1-weighted image (**c**) at the distal portion of the proximal phalanges. The horizontal white bar in **a** indicates the level of transversal slices (**b**, **c**). Sagittal fs proton density-weighted image of the third digit (**d**). **a** Increased signal at the collateral ligaments and synovitis of the proximal interphalangeal (PIP) joint of the third digit (white arrow). Periarticular inflammation around the PIP joint and the body of the proximal phalanx of the third digit (arrowhead). **b**, **c** Extensive flexor tenosynovitis (asterisk) and periarticular inflammation in the subcutaneous tissues (arrowhead) alongside thickened flexor tendon pulleys (arrow). **d**, **e** Representative sagittal T1-weighted images of the MCP, PIP, and DIP joints of the 3rd digit. Following iv contrast administration and appropriate delay of 40 min, **a** gives the morphological T1 map, while **b** gives the corresponding parameter map with dGEMRIC values [ms] overlaid. Note the significant decrease in dGEMRIC indices of the PIP joint as compared to the MCP joint. Also, decreased dGEMRIC indices of the volar aspect of the DIP joint

**Fig. 2:**
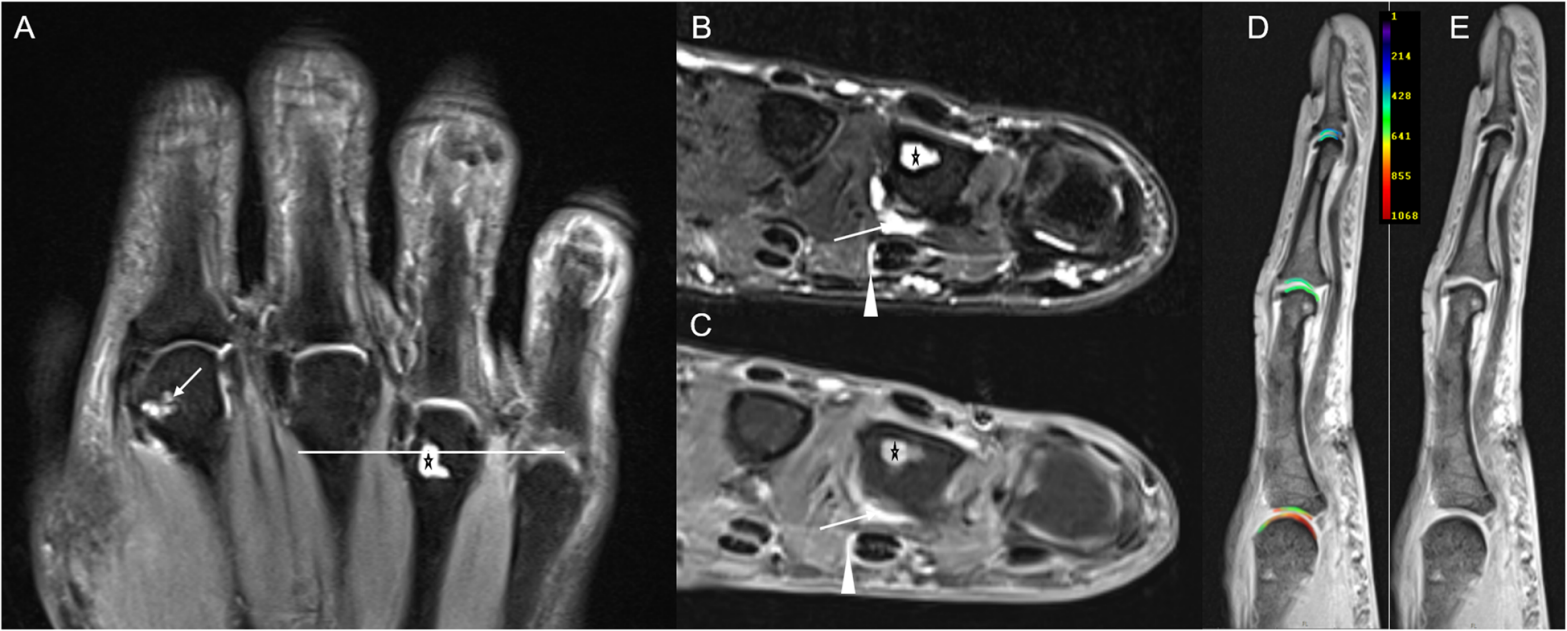
Right hand of a 57-year-old female with PsA (disease duration 46 months). Coronal STIR of digits 2–4 (**a**), transversal T2w fs (**b**), and corresponding contrast-enhanced T1w fs images (**c**) of the metacarpophalangeal (MCP) level of digits 2–4. The horizontal white bar in **a** indicates the level of transversal slices (**b**, **c**). **a** Bone erosions at the metacarpal head of the 2nd (arrow) and 4th (asterisk) digits. **b**, **c** Bone erosion at the fourth metacarpal head (asterisk), synovitis (arrow), and subtle flexor tenosynovitis (arrowhead) at the MCP joint level of the 4th digit. **d**, **e** Representative sagittal T1-weighted images of the MCP, PIP and DIP joints of the 3rd digit. Following iv contrast administration and appropriate delay of 40 min, **d** gives the morphological T1 map, while **e** gives the corresponding parameter map with dGEMRIC values [ms] overlaid. No marked decrease of dGEMRIC indices is seen

Typical disease-related joint changes are visible in Figs. 1 and 2.

### Structural assessment of the joint cartilage: TCT

Descriptive analysis of the TCT of MCP, PIP, and DIP joints 2–5 are given in Table 3. Measurements of TCT were consistent among the ulnar, central, and radial aspects of each joint (e.g., PIP level of digits 2–5: ulnar 0.71 ± 0.23 mm, central 0.71 ± 0.26 mm, radial 0.69 ± 0.23 mm; *p* = 0.989, 0.830, and 0.751, respectively). Overall, the more peripheral the joint is, the thinner its cartilage layers are (MCP 1.07 ± 0.30 mm, PIP 0.71 ± 0.23 mm, DIP 0.57 ± 0.22 mm; *p* < 0.001 and *p* = 0.024).

### Compositional assessment of the joint cartilage: dGEMRIC indices and reliability testing

For the MCP, PIP, and DIP joints 2–5, the descriptive analysis of dGEMRIC indices is displayed in Table 4. The more peripheral the joint is, the smaller the mean dGEMRIC indices are: MCP 533 ± 148 ms, PIP 404 ± 119 ms, and DIP 393 ± 131; *p* = 0.08, 0.07, and 0.968, respectively. Overall, dGEMRIC indices ranged from 157 to 951 ms. AICC was 0.96, and sICC was 0.90 (*p* < 0.001).

**Table 4:** Descriptive analysis of dGEMRIC indices [ms] of the MCP, PIP, and DIP joints 2–5 in PsA patients; for each region, the mean ± standard deviation (SD), the median, and the range (minimum and maximum) are presented. Means were compared by one-way ANOVA and a post hoc Scheffé test. *p* values < 0.05 were considered significant and are given in bold

dGEMRIC indices					
	Overall	MCP	PIP	DIP	*p* value
Mean	431	533	404	393	
SD	152	148	191	131	MCP vs. PIP **0.008**
Median	426	550	413	349	MCP vs. DIP **0.007**
Minimum	157	259	191	157	PIP vs. DIP 0.968
Maximum	951	951	679	783	

### Correlation of compositional and structural cartilage measures as well as PsAMRIS

The correlations of compositional (i.e., dGEMRIC indices) with structural cartilage measures (i.e., TCT) as well as the PsAMRIS sum score and sub-scores (i.e., flexor tenosynovitis, extensor tenosynovitis, synovitis, periarticular inflammation, bone edema, bone erosion, and bone proliferation) for the individual joint levels are shown in Table 5. Significant correlations between GEMRIC indices and the TCT of all joint levels were found (*τ* = 0.43, *p* < 0.001), yet correlations were only significant for the MCP joint level (*τ* = 0.51, *p* = 0.015), while for the PIP and DIP, no significant correlations were found between dGEMRIC indices and TCT (0.04 ≤ *τ* ≤ 0.41, *p* ≥ 0.051).

**Table 5:** Correlation between compositional cartilage measures (i.e., dGEMRIC indices) and structural cartilage measures (i.e., total cartilage thickness, TCT) as well as semi-quantitative measures of joint inflammation, osteoproliferation, and destruction. Correlations were assessed for the metacarpophalangeal (MCP), proximal interphalangeal (PIP), and distal interphalangeal (DIP) joint levels of fingers 2–5. Psoriatic arthritis magnetic resonance imaging score (PsAMRIS) and PsAMRIS sub-scores are synovitis, flexor tenosynovitis, extensor tenosynovitis periarticular inflammation, bone erosion, bone edema, and bone proliferation. *p* values < 0.05 were considered significant and are given in bold. *τ* = Kendall tau correlation coefficient

dGEMRIC	Overall		MCP		PIP		DIP	
	*τ*	*p*	*τ*	*p*	*τ*	*p*	*τ*	*p*
TCT	0.43	**< 0.001**	0.51	**0.015**	0.04	0.826	0.41	0.051
PsAMRIS	− 0.3	0.139	− 0.5	**0.012**	− 0.33	0.102	− 0.43	**0.029**
Flexor tenosynovitis	− 0.34	0.098	− 0.24	0.243	− 0.4	**0.049**	− 0.32	0.109
Extensor tenosynovitis	− 0.31	**< 0.001**	− 0.33	**0.001**	− 0.31	**0.002**	− 0.32	**0.002**
Synovitis	− 0.4	**0.048**	− 0.56	**0.006**	− 0.45	**0.094**	− 0.32	0.109
Periarticular inflammation	− 0.61	**0.003**	− 0.47	**0.022**	− 0.72	**< 0.001**	− 0.37	0.072
Bone erosion	− 0.20	0.320	0.06	0.761	− 0.30	0.880	− 0.37	0.070
Bone edema	− 0.13	0.543	− 0.04	0.846	− 0.17	0.391	− 0.29	0.143
Bone proliferation	0.01	0.954	0.04	0.865	− 0.07	0.732	< 0.01	1

A range of significant negative correlations were determined for dGEMRIC indices and PsAMRIS sum scores as well as acute-inflammatory sub-scores (e.g., PsAMRIS sum score [DIP joint level]: *τ* = − 0.43, *p* = 0.029; flexor tenosynovitis [PIP joint level]: *τ* = − 0.4, *p* = 0.049; extensor tenosynovitis [all joint levels]: *τ* = − 0.31, *p* < 0.001; synovitis [all joint levels]: *τ* = − 0.4, *p* = 0.048; [MCP joint level] *τ* = − 0.56, *p* = 0.006; and periarticular inflammation [PIP joint level]: *τ* = − 0.72, *p* < 0.001). No significant correlations were found between the dGEMRIC indices and bone erosion, edema, or proliferation.

## Discussion

The most important finding of the present study is that proteoglycan loss as assessed by dGEMRIC indices is associated with periarticular inflammation, synovitis, and flexor and extensor tenosynovitis in PsA, but not with bone erosion or proliferation.

Previous studies have already demonstrated that synovitis—a valid marker of acute inflammation—is highly associated with proteoglycan loss in patients with RA at the MCP joint level [29–31]. In our cohort of PsA patients, we found that this association extends beyond intra-articular synovitis to involve periarticular inflammation and flexor and extensor tenosynovitis. These findings support the commonly accepted concept of synovitis being a trigger of cartilage damage by releasing catabolic enzymes that directly affect the cartilage structure and composition by targeting proteoglycans [36]. Even though the pertaining concepts of cartilage damage in inflammatory arthritis are derived from RA-related studies, similar mechanisms of cartilage damage may be relevant for PsA, too, due to shared features of both disease entities, i.e., synovitis and bone marrow edema, despite distinct pathophysiological differences [37]. McGonagle et al. demonstrated that supposedly, bare areas at the MCP joints are regularly coated with joint cartilage at the entheseal insertion sites (collateral ligaments), and hence, cartilage disintegration and eventual damage in those areas are necessary prior to the emergence of (bone) erosions [22]. As PsA is considered an entheseal-driven disease, a comparable mechanism seems plausible [38]. This is further supported by our findings of close correlations of dGEMRIC indices and flexor and extensor tenosynovitis as well as periarticular inflammation—all three very common features of PsA—that suggest that proteoglycan loss and inflammatory arthritis are indeed concomitant phenomena in PsA [7, 39–41]. Additionally, of all acute inflammatory imaging markers, periarticular inflammation showed the strongest correlation with decreased dGEMRIC indices. As periarticular inflammation is the morphological correlate of dactylitis, the hallmark feature of PsA [42–44], this finding indicates once again the close association of acute inflammation and early, i.e., pre-morphological, cartilage damage. Clinical practice and associated research strongly suggest that this imaging finding is of great relevance to the joint’s long-term health with significant therapeutic and prognostic implications: Aletaha et al. demonstrated that cartilage damage is more clearly associated with an irreversible physical disability than bone destruction in patients with RA [45]. These clinical observations were later confirmed in a chronic inflammatory erosive animal model of RA, where the gait parameters were associated with inflammation-mediated joint pathologies and cartilage damage was identified as the main determinant of progressive functional impairment [46]. Even though research on cartilage involvement in PsA is sparse, especially in terms of imaging, studies on cartilage-related biomarkers in PsA such as COMP, OPG, C-terminal cross-linking telopeptide of type II collagen, matrix metalloprotein-3 (MMP-3), and the soluble receptor activator of nuclear factor-κB ligand (sRANKL) indicate an involvement of the cartilage in the disease in all disease stages [47–49]. Future studies that bring together imaging and serological biomarkers may further enhance our understanding of cartilage degradation in PsA. However, cartilage damage is not yet included in the OMERACT PsAMRIS, a validated tool for disease detection and monitoring, as opposed to its RA-related equivalent RAMRIS, where cartilage damage is taken into account by the evaluation of joint space narrowing [12, 18]. If the therapeutic and prognostic implications of cartilage damage in PsA are confirmed, which is not yet the case, thoughtful reconsideration of the scoring system seems indicated.

Furthermore, in RA, compositional MRI techniques have been used for the evaluation of treatment response [30], which could be a potential application in PsA, too. In addition, since studies showed that cartilage matrix components in the serum are predictive of disease progression and outcome [26], the predictive potential of compositional MRI techniques of the cartilage could also be of interest for future research in PsA. Since proteoglycan loss can be reversible and precedes more severe and potentially irreversible structural and other compositional changes in the cartilage, these findings support the concept of early comprehensive (and anti-inflammatory) treatment regimens of PsA and emerging T2T strategies for a better clinical outcome [4, 10, 50]. However, caution is warranted before interventional studies have proven the superiority of a MRI-based T2T concept in PsA since similar studies have failed in RA [13].

As opposed to acute inflammatory changes, we found no significant association of osteodestructive or osteoproliferative changes with low dGEMRIC indices in our patient cohort. These findings are well in line with the recent literature data that indicated that bone marrow edema, but not bone erosion, is correlated with changes of cartilage composition as detected by dGEMRIC in patients with RA [29]. Both bone erosions and bone proliferations are signs of chronic bone changes and hence the result of long-standing inflammation.

In addition, we found significant positive correlations for TCT and dGEMRIC indices, yet only for the MCP joint level. For the PIP and DIP joint levels, no significant correlations were determined with the dGEMRIC indices. Although the exact reason for these discrepancies remains speculative, a possible explanation may involve constitutively different proteoglycan levels in the cartilage layers along the digits or higher PsA-associated proteoglycan loss with largely preserved cartilage thickness in the MCP joints than in the PIP and DIP joints.

In future studies, comparative analyses of cartilage involvement in different forms of arthritis, e.g., RA, hand osteoarthritis (HOA), erosive HOA, and PsA, by the state-of-the-art imaging techniques such as high-resolution morphological and compositional MRI techniques would be a scientifically interesting and clinically relevant field of research. A clearer understanding of the different pathomechanisms may contribute to an improved differentiation of “borderline cases,” even beyond the often sought differentiation of RA vs. PsA. These aspects may help in diagnostically distinguishing between PsA, HOA, and EHOA based on the different patterns of local inflammation and subsequent erosions [51–55].

Our study has limitations. First, we only considered a small study population of PsA patients in a cross-sectional design. Therefore, our findings have to be confirmed by future studies including larger numbers of PsA patients and, potentially, longitudinal study designs, allowing further in-depth analysis of potential associations of early cartilage changes and the development of bone erosions and proliferations over time. Second, even though absolute dGEMRIC values are obtained in the present and other studies, substantial variability in the dGEMRIC protocols limits the generalizability and comparability of our findings. Third, dGEMRIC may be prone to quantification inaccuracy, for example, in the presence of hyperperfusion [56]. Therefore, particular care was taken to delineate and place ROIs as precise and accurate as possible to exclude artifacts and surrounding structures. Fourth, our study population consisted of patients with long-standing PsA. Considering that total joint damage, including damage to both the bone and cartilage, is the result of long-standing and not only acutely present inflammation, the long disease duration certainly influenced the association of dGEMRIC indices and PsAMRIS. Therefore, it remains unknown, whether dGEMRIC changes also occur in patients with early PsA, even though studies of serological markers have demonstrated cartilage involvement in all stages of the disease [47, 49]. Fifth, we did not include a healthy control group to our study to investigate the correlation of absent inflammatory changes and high dGEMRIC indices. However, due to the recent discoveries regarding gadolinium-based contrast agents and their potential risks, the inclusion of healthy volunteers was not justified by ethical standards [57]. In future studies, compositional MRI is needed which does not rely on the intravenous application of contrast agents.

## Conclusion

In conclusion, we found low dGEMRIC indices in the cartilage as an indication of proteoglycan loss to be strongly related to acute inflammatory changes, especially synovitis, periarticular inflammation, and flexor tenosynovitis, but not to bone erosion or proliferation in PsA patients. These findings further illustrate in vivo concepts of the pathophysiology in PsA. Further research on the role of cartilage damage (and its pre-morphological precursors) in PsA is warranted to improve the detection and monitoring of PsA in comprehensive efforts to prevent functional disabilities and potentially to predict long-term outcomes.

## Data Availability

The datasets used and/or analyzed during the current study are available from the corresponding author on reasonable request.
